# BCG Scar Reactivation During Influenza Infection in the Absence of Kawasaki Disease: A Primary Care Case Report

**DOI:** 10.1002/ccr3.73116

**Published:** 2026-07-07

**Authors:** Dilara Canbay Özdemir, Oğuzhan Alp

**Affiliations:** ^1^ Department of Family Medicine, Faculty of Medicine Ordu University Ordu Türkiye

**Keywords:** BCG scar reactivation, BCG vaccine, child, immune activation, influenza B virus, mucocutaneous lymph node syndrome

## Abstract

BCG scar reactivation is classically associated with Kawasaki disease but may also occur during immune activation caused by infections. We report a 26‐month‐old boy with laboratory‐confirmed influenza B who developed transient erythema and swelling at a previous BCG vaccination scar without clinical features of Kawasaki disease or MIS‐C. The scar reaction regressed with symptomatic treatment and oseltamivir as influenza symptoms improved. This case highlights that BCG scar reactivation is not specific to Kawasaki disease and should be interpreted within the full clinical context to avoid unnecessary investigations while maintaining appropriate follow‐up.

## Key Clinical Message

A BCG scar that becomes reactivated is not a definitive indicator of Kawasaki disease. Temporary redness and swelling may occur at the scar site during viral infections like influenza without systemic vasculitis. Influenza virus infection can trigger a temporary immune response in previously sensitized BCG scar tissue.

## 1 Introduction

Bacillus Calmette–Guérin (BCG) vaccine is a vaccine developed against *Mycobacterium tuberculosis* , the etiological agent of tuberculosis [1]. According to the national childhood vaccination schedule of the Ministry of Health of the Republic of Turkey, it is administered intradermally to infants who have completed 2 months of age [2]. Pustules and ulceration may be seen at the injection site approximately 2 weeks after vaccination, and they heal after a few weeks, leaving a scar [3]. This scar is usually permanent and is not expected to reactivate. However, the development of erythema and swelling in the BCG scar area has been most frequently associated with Kawasaki disease (KD) in the literature [4, 5]. In recent years, this finding has also been observed after Multisystem Inflammatory Syndrome in Children (MIS‐C), COVID‐19, and influenza vaccine administrations [6, 7, 8]. However, reactivation of the BCG scar after viral infections without KD is extremely rare [9, 10].

This case report presents a child who developed redness and swelling at the BCG injection site following an influenza infection.

## 2 Case History/Examination

A 26‐month‐old boy presented to the family medicine clinic with complaints of fever, runny nose/nasal congestion, cough, and redness/swelling at the BCG injection site, all of which had been ongoing for approximately 2 days. The family reported that the patient's fever reached a peak of 39.2°C and subsided after cold compresses and antipyretic treatment.

The patient received the BCG vaccine at 2 months of age as part of the routine postnatal vaccination schedule, and a normal scar developed. It was learned that the patient had no previous hospitalizations, had received all vaccinations according to the national vaccination schedule, and there was no consanguineous marriage. A review of the patient's past laboratory results revealed no pathological findings.

**FIGURE 1:**
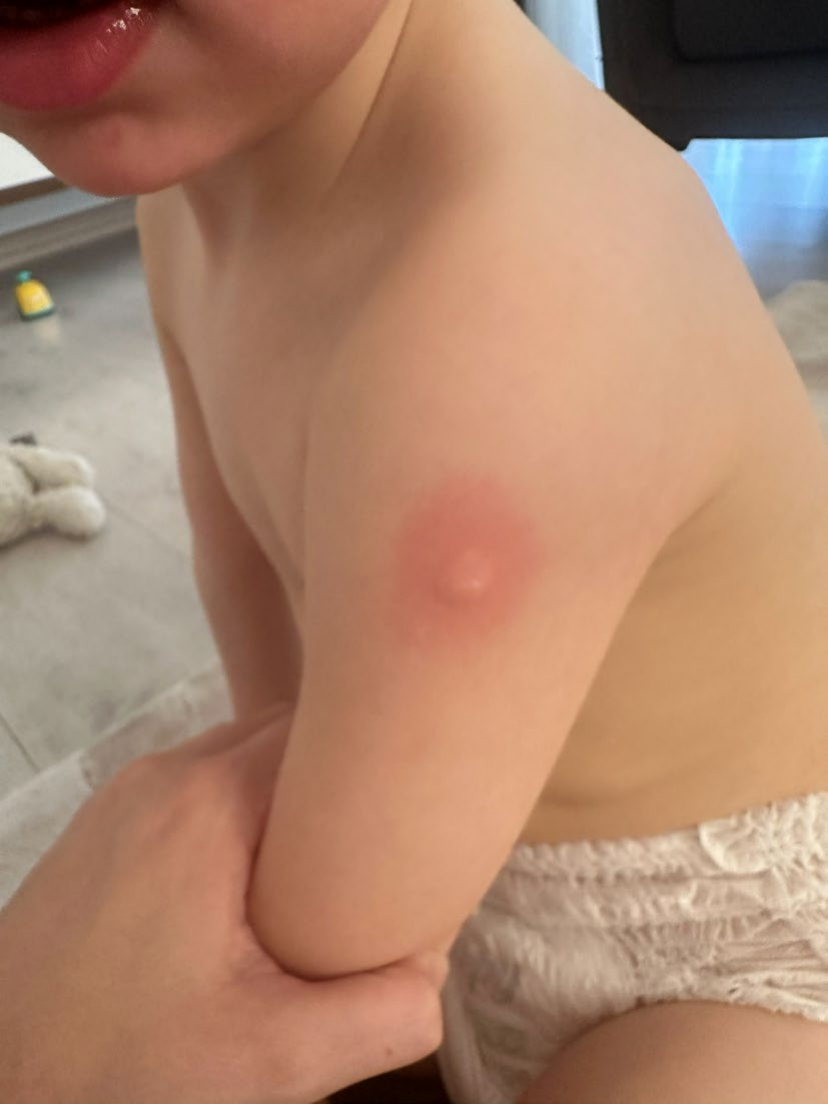
Bacillus Calmette–Guérin Scar reactivation.

Body temperature was 36.7°C. The BCG injection site on the upper left arm was red, slightly swollen, firm, and tender (Figure 1). Skin examination revealed no rash, erythema, or peeling on the palms and soles of the feet. Lip and oral mucosal examinations were normal. The oropharynx was hyperemic, and there was postnasal drip. The patient showed no signs suggestive of Kawasaki disease, such as mucocutaneous findings, cervical lymphadenopathy, or coronary artery involvement.

## 3 Differential Diagnosis, Investigations and Treatment

In the differential diagnosis, KD, MIS‐C, local bacterial skin infection (cellulitis or lymphangitis), hypersensitivity reactions, and mechanical irritation of the scar were included. The lack of persistent high‐grade fever, mucocutaneous changes, conjunctival injection, cervical lymphadenopathy, extremity changes, and other systemic manifestations made KD less likely. There were no clinical manifestations of MIS‐C or bacterial soft tissue infection.

**FIGURE 2:**
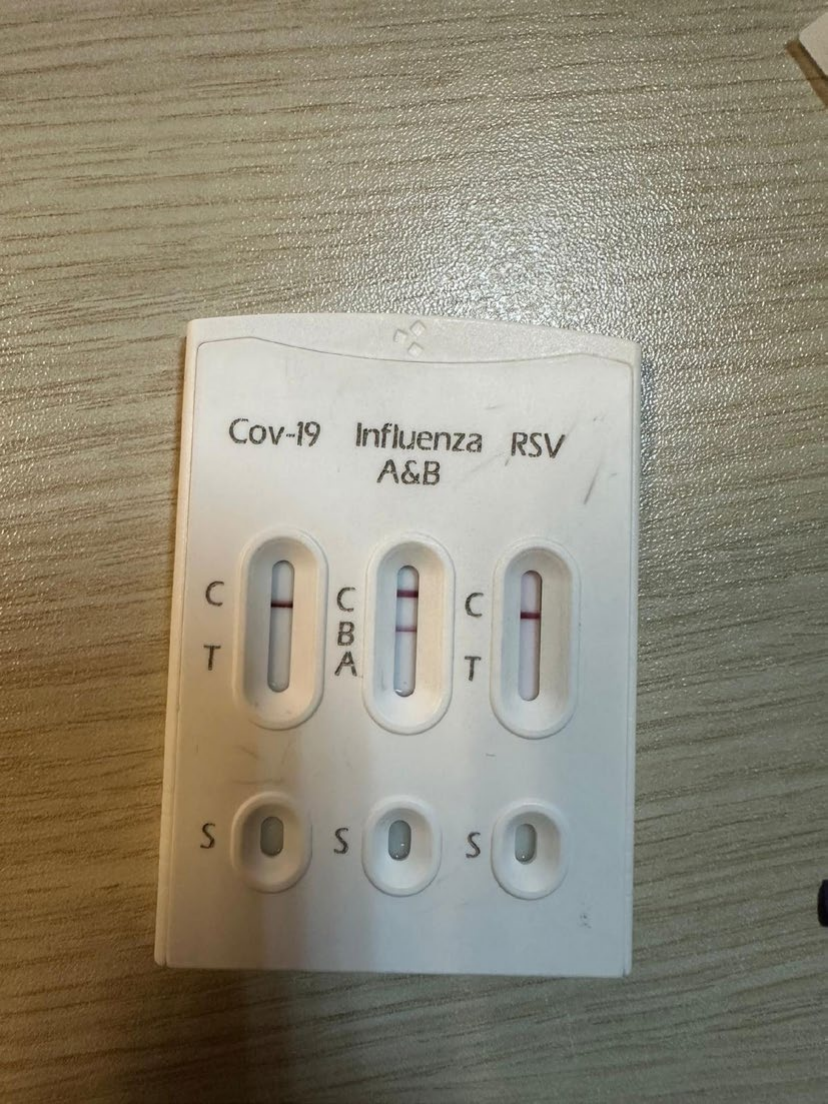
Rapid antigen test result.

Laboratory studies showed hemoglobin of 12.1 g/dL (within age‐related normal limits), C‐reactive protein of 9.3 mg/L (mildly elevated), leukocyte count of 4300/mm^3^ (leukopenia), lymphocyte count of 1000/mm^3^ (lymphopenia), lymphocytes 2400/mm^3^, monocytes 300/mm^3^, and platelet count of 333,000/mm^3^ (normal). The hematologic findings were suggestive of a viral infection. A rapid antigen test done on a nasopharyngeal swab was negative for COVID‐19 and respiratory syncytial virus but positive for influenza B virus (Figure 2).

Since the patient did not have clinical features of KD and no evidence of coronary involvement, further cardiac workup was unnecessary. The patient was treated symptomatically with antipyretics and hydration. Oseltamivir was started based on age‐related dosing recommendations.

## 4 Conclusions and Results (Outcome and Follow‐Up)

The patient began to demonstrate clinical improvement a few days after the initiation of symptomatic management and oseltamivir. The erythema and induration at the BCG scar site also regressed in accordance with the resolution of systemic influenza symptoms. There were no additional mucocutaneous manifestations, fever, or features of Kawasaki disease. The BCG scar was found to be back to normal at the 15‐day outpatient follow‐up without any complications.

## 5 Discussion

This case is noteworthy because it demonstrates that erythema and swelling can develop at the BCG scar site simultaneously with influenza infection, even without KD. A review of the literature revealed no case of BCG reactivation in a child following influenza infection. BCG scar reactivation is a finding classically associated with KD and is considered one of the helpful clinical signs in the diagnosis of KD [11, 12]. However, in recent years, transient reactivations of BCG scars have also been reported following vaccinations and various viral infections such as measles and herpes virus [6, 7, 9, 10]. To the best of our knowledge, there are no previous cases that have mentioned the reactivation of BCG scars in relation to laboratory‐proven influenza infection but not KD.

In the present case, the lack of persistent fever, mucocutaneous manifestations, thrombocytosis, and coronary manifestations makes KD unlikely. The parallel regression of scar inflammation with the improvement of viral symptoms further supports the interpretation of a transient, infection‐triggered immune response rather than a vasculitis process.

From a pathogenesis perspective, several hypotheses are put forward [13, 14]: (i) the “trained immunity” effect of BCG on macrophages and natural killer cells; (ii) the increased pro‐inflammatory cytokines and chemokines during viral infection facilitating local inflammation around the scar; (iii) the already sensitized cellular response to persistent mycobacterial antigens in BCG scar tissue exceeding the threshold with systemic inflammation. These mechanisms may help explain why transient, self‐limiting reactivations can occur in BCG scars even without KD in the clinic.

Because influenza virus infection induces a robust innate antiviral immune response—including monocyte/macrophage activation, natural killer cell responses, type I interferon signaling, and the release of pro‐inflammatory cytokines and chemokines—it represents a biologically plausible trigger for BCG scar reactivation [15]. In children previously vaccinated with BCG, trained innate immune cells and antigen‐specific memory responses may retain the ability to mount an enhanced inflammatory response when exposed to subsequent systemic infection stimuli [13, 14]. Therefore, the inflammatory environment created during influenza infection may lower the reactivation threshold of pre‐established immune responses in the BCG scar region, where residual mycobacterial antigens or locally sensitized immune cells may persist [13, 14, 15]. From this perspective, the BCG scar reactivation in the present case can be interpreted not merely as a coincidental local skin finding, but as a visible manifestation of immune activation in previously primed tissue triggered by a systemic infection. The resolution of the scar reaction concurrent with improvement in influenza symptoms further supports the notion that this is a transient and self‐limiting feature rather than one associated with Kawasaki disease‐related vasculitis.

In the differential diagnosis, KD should be considered, along with MIS‐C, local skin infection (cellulitis/lymphangitis), hypersensitivity reactions, and rarely trauma/irritation to the scar area. In this case, (i) the absence of mucocutaneous findings, (ii) the lack of clinical evidence suggesting coronary artery involvement, (iii) the reaction occurring simultaneously with viral symptoms and disappearing with the regression of symptoms, and (iv) the observation of a pattern consistent with viral infection, such as leukopenia and lymphopenia in laboratory tests, rule out KD and bacterial etiologies.

The clinical implications are as follows:

1. Redness and swelling in a BCG scar are not, by themselves, specific in favor of KD; the possibility of a transient reactivation associated with infection should be kept in mind, especially when seen during viral upper respiratory tract infections.
2. Clinical integrity is essential to avoid unnecessary investigations and treatments; if there are no systemic findings supporting KD, close monitoring, symptomatic treatment, and management of the underlying infection may be sufficient in most cases.
3. The spontaneous regression of scar reactivation as systemic symptoms improve following antiviral treatment (e.g., oseltamivir) supports the secondary and self‐limiting nature of this condition.

Clinically, inflammation at a BCG scar site should not be regarded as pathognomonic for KD. Careful correlation with systemic findings is essential to avoid misdiagnosis and unnecessary investigations. Recognition of this benign and self‐limiting presentation may support appropriate monitoring and conservative management.

This case report expands the clinical spectrum of BCG scar reactions following infection and raises the possibility that viral infections can induce transient immune activation of previously sensitized scar tissue. While this case report cannot establish causality, further case reports and research studies are required to better understand the underlying immunological mechanisms. This case report also emphasizes the importance of clinical follow‐up.

## Author Contributions

**Dilara Canbay Özdemir:** conceptualization, investigation, methodology, project administration, resources, supervision, writing – review and editing. **Oğuzhan Alp:** conceptualization, investigation, methodology, writing – original draft.

## Funding

The authors have nothing to report.

## Ethics Statement

The authors have nothing to report.

## Consent

Written informed consent for publication of clinical information and images was obtained from the patient's parents.

## Conflicts of Interest

The authors declare no conflicts of interest.

## Data Availability

No datasets were generated or analyzed during the current study. All relevant clinical information is included within the article. Further details are not publicly available due to patient privacy considerations.
